# Chlorine Reduction Kinetics and its Mass Balance in Copper Premise Plumbing Systems During Corrosion Events

**DOI:** 10.3390/ma12223676

**Published:** 2019-11-08

**Authors:** Ignacio T. Vargas, Javiera M. Anguita, Pablo A. Pastén, Gonzalo E. Pizarro

**Affiliations:** 1 Centro de Desarrollo Urbano Sustentable (CEDEUS), Santiago 7520246, Chile; ppasten@ing.puc.cl; 2 Department of Hydraulic and Environmental Engineering, Pontificia Universidad Católica de Chile, Santiago 7820436, Chilegpizarro@ing.puc.cl (G.E.P.)

**Keywords:** drinking water, premise plumbing, copper corrosion, chlorination, chlorine consumption

## Abstract

Hypochlorous acid has been reported as the main oxidant agent responsible for the corrosion of copper plumbing systems in chlorinated water supplies. However, there is little information about chlorine consumption kinetics in a combined system (i.e., with dissolved oxygen (DO) and free chlorine), as well as its complete mass balance within a copper pipe during stagnation. The results of our experiments using copper pipes filled with synthetic drinking water, with a moderate alkalinity (pH = 7.2; dissolved inorganic carbon = 80 mg as CaCO_3_ /L), and tested under chlorine concentrations from 0 to 8 mg/L, show that chlorine depletion is associated with pipe wall reactions (i.e., copper oxidation and scale formation processes). Free chlorine was depleted after 4 h of stagnation and its kinetic constant depend on the initial concentration, probably due to diffusion processes. Surface analysis including scanning electron microscopy (SEM), energy dispersive spectroscopy (EDS) and total reflection X-ray fluorescence (T-XRF) suggest chlorine precipitation, probably as CuCl. The obtained kinetics of chlorine and DO reduction would be critical for modeling and prediction of corrosion events of copper premise plumbing systems. In addition, our results indicate that the pipe’s surface reactions due to corrosion induces a loss of free chlorine in the bulk water, decreasing chlorine added for disinfection and the subsequent effect on water quality.

## 1. Introduction

Chlorination is the most used method for disinfection worldwide [1]. The disinfection is produced by the chlorine oxidative capacity [2]. Typical levels of free chlorine in drinking water range from 0.2 to 0.5 mg/L [3]. However, to guarantee an adequate disinfection and to maintain a residual chlorine concentration, the actual doses added are usually higher. To achieve this standard, the World Health Organization established chlorine concentrations from 0.2 to 5 mg/L at the point of delivery [3]. In neutral drinking waters, chlorine is commonly found as hypochlorous acid (HOCl), a weak acid that does not significantly affect pH [4]. In spite of this, HOCl is a strong oxidizing agent and must be considered in the cathodic half-reaction of metallic copper oxidation [4].

Copper is a widely-used material for premise plumbing due to its relative resistance to corrosion. However, copper pipes are subjected to suffer corrosion under certain operational conditions [5]. It starts as an electrochemical phenomenon involving two half-reactions, one responsible for the release of copper ions into the water (anodic half-reaction), and one involving the reduction of an oxidizing agent (cathodic half-reaction). In general, the most available electron acceptor in drinking water systems is dissolved oxygen (DO), however, chlorine added for disinfection purposes may also serve as an additional oxidizing agent [6] for copper corrosion.

Depending on its concentration, previous works have reported two distinct effects of chlorine on copper corrosion [4,7,8,9,10,11,12,13,14,15,16]. In moderate to high chlorine concentrations, a direct relationship between chlorine concentration and copper release has been reported [4,9,10,11,12,13,14]. For low and moderate chlorine concentrations (below ~2.6 mg/L), chlorine may prevent microbial influenced corrosion episodes [7,8,15,16]. Based on this evidence, research developed during the 90s recommended chlorine concentrations of ~2 mg/L to control biocorrosion episodes and to secure disinfection [4,8]. However, to the best of our knowledge, the cross-linked processes of copper corrosion and chlorine consumption in drinking water during stagnation periods has not been completely addressed.

Thermodynamic and kinetic evidence support that hypochlorous acid is the main oxidizing agent responsible for copper corrosion in chlorinated water supplies, competing with the available DO at the metal surface as electron acceptor of the corrosion reaction [4,17]. Thermodynamically, the reduction potential of hypochlorous acid is higher than the DO reduction potential under standard conditions. Despite DO and hypochlorous acid losing their oxidizing ability as pH increases, the reduction potential of DO decreases twice as fast as hypochlorous acid [4]. Kinetically, the corrosion rates of copper with either chlorine or DO have been studied [17]. By using cathodic polarization, Cong and Scully showed that chlorine has higher reduction rates than oxygen [17]. Previous studies have also shown that the kinetics of DO and residual chlorine consumption during stagnation follows a first-order kinetics [18,19,20,21,22]. Studies with chlorinated drinking water have demonstrated that free chlorine is mainly responsible for copper corrosion, while DO plays a secondary role during stagnation [4,17]. However, according to the best of our knowledge, previous research has not studied the combined kinetics of DO and chlorine consumption in copper pipes; neither has it accounted the fate of added chlorine (chlorine in the bulk water vs. chlorine in the scales).

The existing literature addressing the effect of chlorine on the corrosion of copper pipes does not consider the combined dynamic interaction between free chlorine and DO under realistic conditions. This could be especially important in experiments with pipes (instead of coupons) tested using actual drinking water conditions (e.g., pH, chlorine, and DIC concentrations, stagnation time) [23,24,25]. Moreover, the existing studies of chlorine consumption in drinking water systems have not coupled “pipe wall” reactions with “bulk” reactions [26,27,28], paying little attention to the fate of chlorine in the system, especially in accounting for the effect of wall reactions.

In consequence, a mechanistic approach is necessary to understand the interaction and processes occurring at the metal-liquid interface, where copper is oxidized and the corrosion by-products control copper leaching and chlorine consumption. In this article, the combined depletion kinetics of chlorine and DO were studied, together with the fate of chlorine via its mass balance during stagnation. Surface analysis and thermodynamic modelling were performed to develop a conceptual model of copper pipe corrosion in chlorinated drinking water systems.

## 2. Materials and Methods

The methodology combines copper pipes experiments using synthetic water with a multi-technique characterization of water chemistry, surface analysis of the corrosion by-products, and modeling to calculate kinetic parameters of DO and chlorine consumption. Experimental data were used to support a proposed conceptual model for copper speciation and chlorine depletion.

Pipe tests were conducted in duplicates, during 8 h of stagnation and using 20 cm copper pipe sections of 1.95 cm internal diameter, type L, and manufactured by MADECO-Chile under ASTM-B88. Additionally, glass graduated cylinders of similar dimensions were used for control stagnation experiments without the “wall effect” (i.e., depletion of chlorine and DO due to copper corrosion). The pipes were filled with synthetic water prepared using MilliQ water, NaHCO_3_ (ACS grade, 99.7% pure, Merck KGaA, Darmstadt, Germany) to adjust a dissolved inorganic carbon (DIC) concentration of 80 mg as CaCO_3_/L and NaClO (available chlorine ≥4%, Sigma-Aldrich, St. Louis, MO, USA) to adjust chlorine concentration from 0 to 8 mg/L. The pH was adjusted to 7.2 with HNO_3_ (65%). The temperature was controlled during the stagnation at 25 °C. The tested pipes were preconditioned according to the procedure presented in our previous work [29]. DO concentration was continuously measured every 15 minutes using a Luminescence Dissolved Oxygen (LDO) sensor connected to a HQ40d multimeter (HACH Company, Loveland, CO, USA). The LDO sensor was inserted at one end of the pipe (the opposite end closed with a rubber stopper) following the experimental set-up described in our previous work [29]. To remove DO and ensure anoxic conditions, tested pipes were flushed with N_2_/CO_2_ (80:20).

### 2.1. Water Chemistry

Water samples were taken from the pipes trying to avoid flushing effects and corrosion by-products detachment. The pH was determined by a Thermo Orion 420A pH meter with a ROSS Sure-flow Semi-Micro, epoxy body pH electrode model Orion 8175 (ORION, Cambridge, MA, USA). Chloride was measured using an ion selective electrode (ISE) model Orion 96-17 (ORION, Cambridge, MA, USA) connected to a Thermo Orion 720A meter. Chlorine was measured using the diethyl-p-phenylene diamine (DPD) method (HACH #8167) with a HACH DR/2010 portable spectrophotometer, providing an analytical window of 0.02–2.00 mg/L that has been used to determine chlorine in several previous corrosion studies [4,7,13,26,30]. Water samples for measurement of copper release were acidified to pH < 2 with concentrated nitric acid, and stored at room temperature to dissolve copper particles [24]. Copper released was measured using the bicinchoninate method (HACH #8506) with a HACH DR/2010 portable spectrophotometer, with an analytical window of 0.04–5.00 mg/L that also has been used in previous corrosion studies to determine total and dissolved copper concentrations [9,18,31,32]. Additionally, the method was tested using a Varian SpectrAA 800 flame atomic absorption spectrometer, showing excellent agreement for concentrations above 0.1 mg/L [18].

According to previous studies, DO and chlorine consumptions were fitted to first-order kinetics estimating the specific consumption rates, *K_O2_* and *K_Cl2_*, in Equations 1 and 2 [18,19,20,21,22]. Nevertheless, each initial concentration was fitted separately. The fits were performed using the minimization of weighted root mean squared error [33].

(1) $${\left[O_{2}\right]}_{\left(t\right)}=\;{\left[O_{2}\right]}_{\left(t=0\right)}e^{-K_{O_{2}}\cdot t}$$

(2) $${\left[Cl_{2}\right]}_{\left(t\right)}=\;{\left[Cl_{2}\right]}_{\left(t=0\right)}e^{-K_{Cl_{2}}\cdot t}$$

${\left[O_{2}\right]}_{\left(t\right)\;}$: Oxygen concentration measured in the bulk in time *t*
${\left[Cl_{2}\right]}_{\left(t\right)\;}$: Chlorine concentration expressed like molecular chlorine
$K_{O_{2\;}}$: Specific consumption rates of oxygen
$K_{Cl_{2}\;}$: Specific consumption rates of chlorine

### 2.2. Surface Analysis

After the experiments, 1 × 1 cm coupons were extracted from the pipes for surface analysis with SEM, EDS, and T-XRF. SEM and EDS were used to study morphology and elemental composition of external/superficial films of corrosion by-products formed on the copper surface. 

A scanning electron microscope (1420VP, LEO Electron Microscopy Ltd., Cambridge, UK) coupled to an Oxford 7424 solid-state detector was used to obtain the micrographs and an estimation of the elemental composition. Copper coupons extracted from one set of tested pipes were embedded in epoxy resin from Buehler [34]. Later, the resin was removed from the inner part of the pipe, extracting with it the thin layer of corrosion by-product formed on the pipe. The thin layer of corrosion by-products attached to the resin was analyzed by EDS (1420VP, LEO Electron Microscopy Ltd., Cambridge, UK) to identify the presence of elemental chlorine in the extracted scales.

Grazing incidence X-ray diffraction (GI-XRD, D8, Bruker, Karlsruhe, Germany) was used to characterize the upper layer of corrosion scales. Solid phases were identified using a Bruker D8 Advance diffractometer with a 40 kV/30 mA copper cathode and a Sol-X detector. The experiments were performed continuously from 5° to 90° with a scan step size of 0.02 [29].

A T-XRF (S2 PICOFOX, Bruker, Karlsruhe, Germany) was used to determine the presence of Cu-Cl scales on deeper layers of the film formed on the inner surface of the pipe. Two different protocols for removing scales from copper coupons were used. The first protocol consisted in sonication by using an ultrasonic water bath (model Elmasonic S 10 H, Elma, Singen, Germany) at 37 kHz for 60 min, and subsequent centrifugation for 60 min at 11,400 g (model Eppendorf MiniSpin, Eppendorf AG, Hamburg, Germany). Samples of 10 µL were pelleted directly to the T-XRF sample holder to quantify the mass of chlorine in the pipe inner-surface. The second protocol is similar, but adding surface scraping after sonication for removing corrosion by-products not extracted by sonication.

### 2.3. Thermodynamic Modeling

Thermodynamic calculations were performed using the software ChemEQL 3.0 (version 3.0, Swiss Federal Institute for Environmental Science and Technology, Dübendorf, Switzerland) [35] to estimate the solubility of corrosion by-products under the experimental conditions and to build a Pourbaix diagram of the copper solid phases. ChemEQL 3.0 has been previously used to model water chemistry equilibrium conditions and precipitation-dissolution processes [36,37,38]. Thermodynamic data for cupric species were obtained from the literature [39,40]. The soluble copper concentration can be expressed as the sum of the soluble cupric species, assuming that a solid phase is formed [32,39,41].

## 3. Results and Discussion

The results of this study have been organized in 3 sections: (1) Water chemistry, including DO vs. chlorine consumption, and copper release measurements; (2) surface analysis with SEM, T-XRF, and GI-XRD; and (3) a mass balance for chlorine including water chemistry, surface analysis, and thermodynamic calculations.

### 3.1. Water Chemistry

#### 3.1.1. DO Kinetics

**Figure 1:**
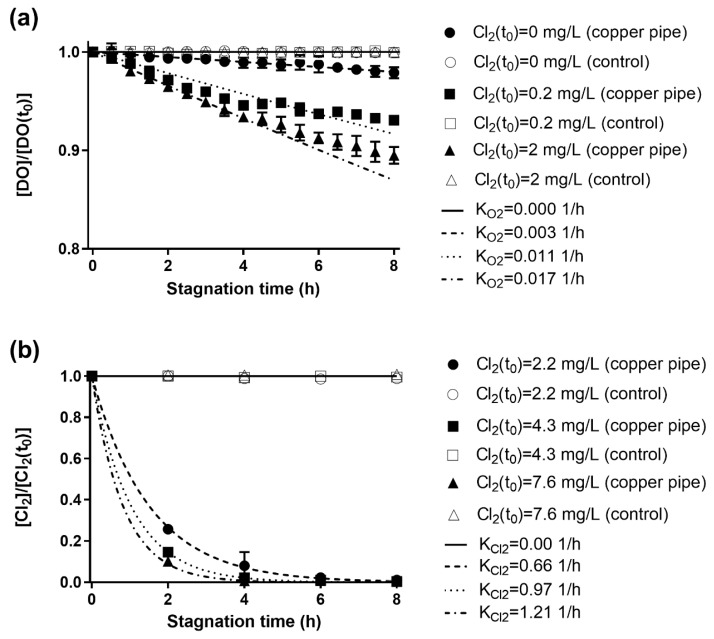
Consumption of dissolved oxygen (DO) and chlorine in new copper pipes during stagnation, the depletion of both oxidizing agents follows a first order kinetics rate law. (**a**) DO consumption during stagnation is significantly affected by the presence of chlorine in the range of concentration expected in drinking water (initial DO was 7.66 ± 0.10 mg/L). (**b**) Chlorine is depleted in about 4 h due to corrosion; no difference was observed in experiments with DO (initial DO was 7.48 ± 0.12 mg/L) and without DO (below detection limit of 0.10 mg/L). The error bars represent the standard deviation (n = 2). The control experiments of copper pipes (glass cylinders) are represented by the same (but empty) symbols.

The effect of residual chlorine as an oxidizing agent on the DO consumption is shown in Figure 1a. The fits of DO consumption were performed with Equation 1. For control experiments (glass cylinders), no significant consumption was observed for all different Cl_2_ concentrations (α = 0.05). The estimation of *K_O2_* for Cl_2_ concentration of 0; 0.2; and 2 mg/L were 0.003 ± 0.001; 0.011 ± 0.004; and 0.017 ± 0.003 L/h, respectively. These results show that chlorine affects DO consumption, probably by an increase in the chemical and electrochemical activity at the pipe wall (i.e., copper inner surface) due to Cu^0^ and Cu(I) oxidation and copper-oxide formation via chlorine reduction. Therefore, the DO consumption is consistent with wall reactions, mainly metallic copper oxidation and scale formation reactions, being the DO consumption enhanced by the presence of chlorine.

#### 3.1.2. Chlorine Kinetics

The chlorine consumption followed a first-order kinetics in copper pipes (Figure 1b). No chlorine consumption was observed in the control tests (*K_Cl2_* = 0.00 L/h), supporting the hypothesis that chlorine depletion is associated with wall reactions, mainly metallic copper oxidation and scale formation reactions. In copper pipes, free chlorine was depleted after 4 h of stagnation (consumption >95%), similar to the results obtained by Li et al. [26] in an experiment conducted with copper pipes and tap water with a chlorine concentration of 7.3 mg/L; unfortunately, *K_Cl2_* values were not reported. The estimation of *K_Cl2_* for Cl_2_ concentration of 2.2; 4.3; 7,6 mg/L were 0.66 ± 0.16; 0.96 ± 0.04; and 1.21 ± 0.04 L/h, respectively. Since the obtained values for *K_Cl2_* increased together with Cl_2_ concentration, higher order kinetics (second and third orders) were evaluated (data not shown). However, better fits were not obtained. Assuming that wall reactions are responsible for Cl_2_ consumption, the difference between the obtained *K_Cl2_* with different initial Cl_2_ concentration could be attributed to the Cl_2_ diffusion process from the bulk to the wall. Previous studies suggest an effect of the initial concentration of chlorine on the chlorine consumption rate [19,30], attributing it to other reactants in the bulk water (e.g., ammonia, organic compounds). Interestingly, the effect of wall reactions on chlorine consumption has not been previously addressed and discussed. The ratio between free chlorine and DO consumption rates (*K_Cl2_*/*K_O2_*) was 39 for a chlorine concentration of ~2 mg/L. This result supports the idea that free chlorine is the main responsible for the metallic copper oxidation in chlorinated household drinking water systems.

#### 3.1.3. Copper Release

Although copper release increased with the initial chlorine concentration, our results suggest that for higher chlorine concentration (greater than 4 mg/L) the increment reaches a maximum limited by the solid phase of Cu(II) that controls copper solubility. The current knowledge of copper corrosion thermodynamics proposes that soluble corrosion by-products released into the water from new copper pipes is controlled by the solubility of cupric hydroxide [39,40]. In fact, the solubility of cupric hydroxide under the experimental conditions (pH = 7.2, DIC = 80 mg as CaCO_3_ /L, 25 °C and 8 h of stagnation) was calculated to be 1.8 mg/L (asymptotic upper limit in Figure S1). It must be kept in mind that thermodynamic predictions do not provide information about the kinetics of copper corrosion by-products solubility, and that the thermodynamic equilibrium is not expected at 8 h of stagnation [42]. Thus, it is reasonable to expect copper concentrations lower than 1.8 mg/L, in spite that some particulate corrosion by-products might be detached during sampling by shear stress, slightly increasing total copper measurements [24].

**Figure 2:**
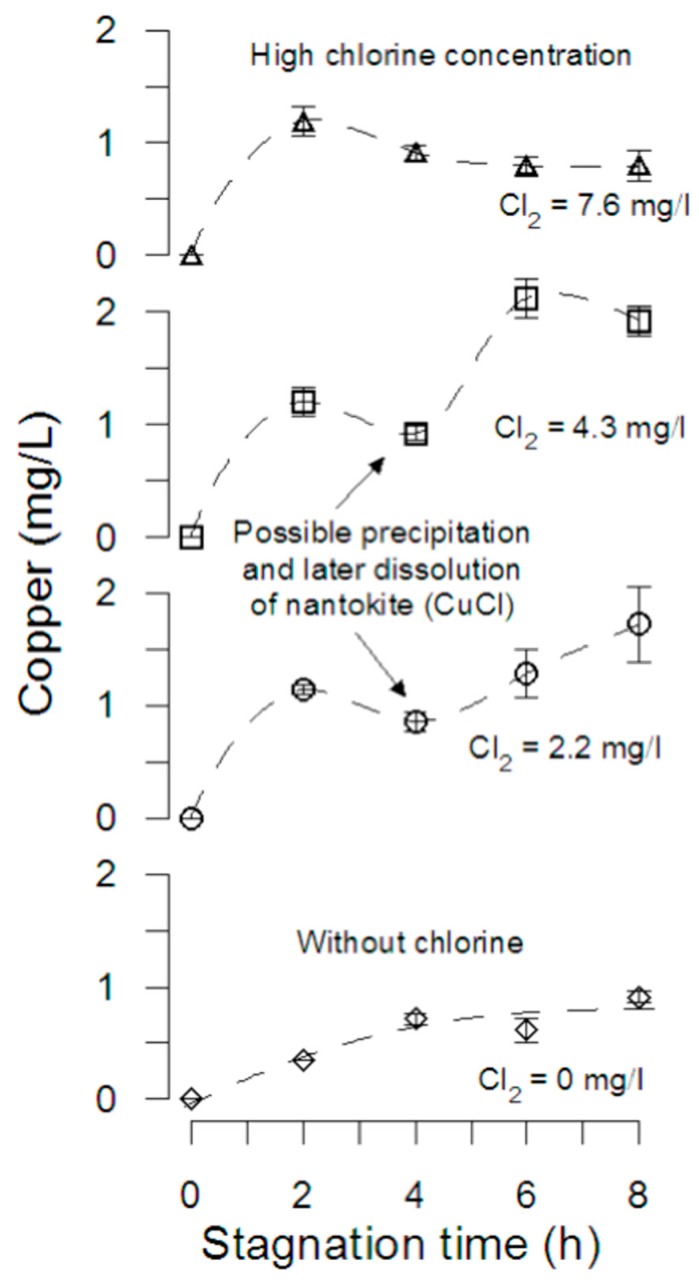
Copper release during stagnation. Pipe tests with different initial concentrations of chlorine. The error bars represent the standard deviation of the measurement (n = 2). Initial DO was 7.45 ± 0.12 mg/L.

Figure 2 shows copper released during 8 h of stagnation with free chlorine concentrations of 0; 2.2; 4.3; 7.2 mg/L. In the absence of free chlorine, the stagnation curve shows an asymptotic increase until a copper concentration of 1 mg/L, probably because the absence of free chlorine to increase the copper corrosion. Although the addition of chlorine increases the amount of copper released after 8 h of stagnation, experiments with free chlorine concentration of 2.2 and 4.3 mg/L show that copper concentration decreased at 4–5 h of stagnation, probably due to nantokite (CuCl_(s)_) precipitation and later dissolution, increasing copper concentration in solution. For the initial two hours, the pipes tested with 7.6 mg/L of free chlorine, the copper concentration increased from 0 to 0.8 mg/L, similar to the 0.91 mg/L measured after 8 h when no chlorine was added, in agreement with the results obtained by Atlas et al. [4] in experiments performed in flasks with copper coupons and tap water adjusted to chlorine concentration of 10 mg/L and pH 8. Hence, an early increase in copper ions released due to changes in corrosion potential induced by high chlorine reduction and local variations in pH (due to the cathodic half-reaction) could result in nantokite precipitation, early malachite (Cu_2_CO_3_(OH)_2_) formation, and subsequent passivation. The speciation of corrosion by-products and thermodynamics will be discussed in detail in Section 3.2 and Section 3.3.

**Figure 3:**
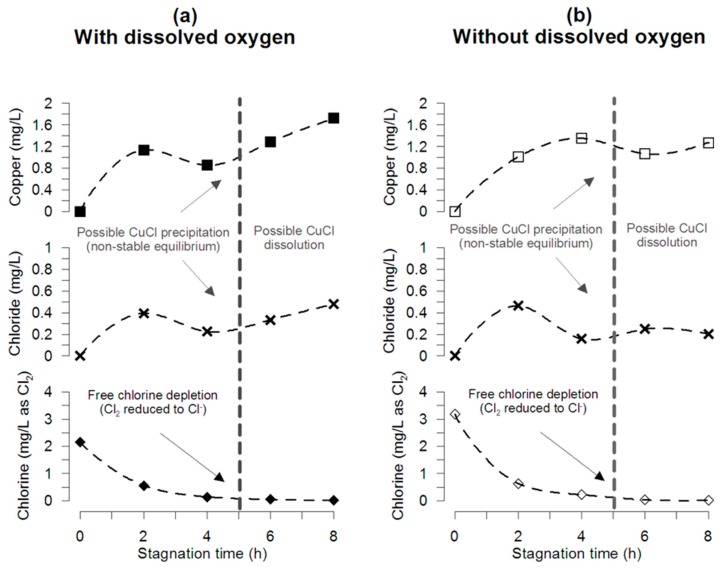
Mass balance during stagnation. A decrease in the copper concentration is reached at 4 h, probably because of Cu-Cl precipitation during the first hour of stagnation. The pipe experiments were conducted using water (**a**) with oxygen (7.59 ± 0.00 mg/L), and (**b**) without oxygen. Interestingly, in both cases, copper and chloride present a minimum concentration at the time that chlorine is almost completely depleted (0.02 ± 0.00 mg/L).

The mass balance of chlorine within the pipe during stagnation for pipe tests at pH 7.2 and 25 °C with and without DO and initial chlorine of 2.2 and 3.2 mg/L is presented in Figure 3a,b, respectively. Even though chloride concentration is significantly lower than the initial free chlorine concentration, results of chloride and copper release show a decrease with a measured local minimum at the time when free chlorine is completely consumed (4–5 h) for the experiments with and without DO. These results agree with the free chlorine consumption presented in Figure 1b, chloride formation in Figure S2, and copper released in Figure 2.

### 3.2. Surface Analysis

SEM-EDS analysis of coupons extracted from copper pipes after 2; 4; 6; and 8 h of stagnation with synthetic water of chlorine concentration of 4 mg/L, DIC of 80 mg as CaCO_3_, and pH 7.2. No significant differences in the morphology of the film formed at different stagnation times were observed during the experiments (Figure S3). No pitting was observed, and a homogeneous film was formed in all cases. EDS analysis reveals an elemental composition of copper, oxygen, and carbon (an average of Cu = 56 ± 6%; O = 4 ± 1%; and C = 40 ± 6%). Additionally, copper coupons exposed for 8 h to different free chlorine concentration (~0; 2; 8 mg/L) were analyzed with SEM-EDS, obtaining similar results (data not shown).

GI-XRD analysis of the inner surface of pipes tested at 4 and 8 mg/L of chlorine confirmed that the outmost layers of corrosion by-products are composed mainly by malachite, and that cupric chlorine-content scales (e.g., atacamite (CuCl_2_·3Cu(OH)_2__(s)_)) are not present (data not shown). Although only malachite was identified as Cu(II) solid phase that is controlling copper solubility, it is important to keep in mind that precipitation of divalent copper oxides as tenorite (CuO) is also expected, and hydrated solid phases with short-range ordering (e.g., cupric hydroxide) are not detected by GI-XRD. For that, complete surface characterization techniques such as X-ray absorption spectroscopy are required [31]. Similar GI-XRD analysis of the inner surface of copper pipes tested with high concentrations of biocarbonate (350 mg as CaCO_3_/L) showed that the surface was composed by cuprite, tenorite, and mainly malachite [29].

**Figure 4:**
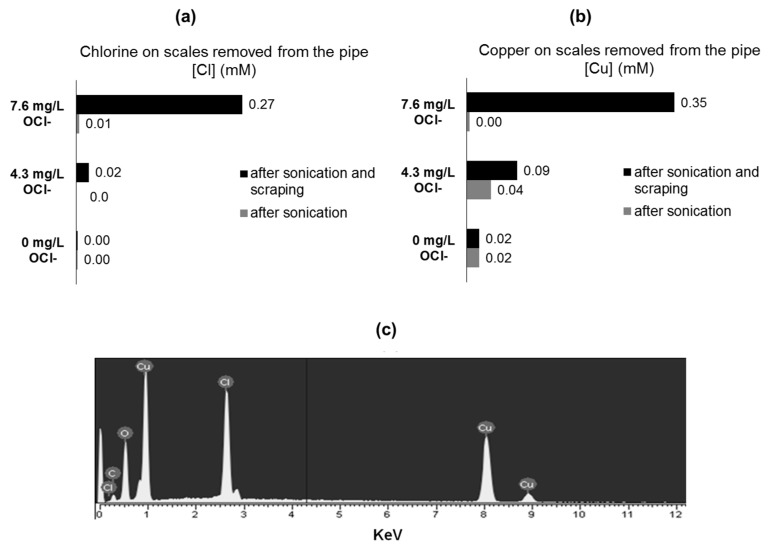
T-XRF measurements of scales removed from the tested copper pipes. Scales removal was done though simple coupon sonication and sonication-scraping (**a**) Chlorine, (**b**) Copper, (**c**) EDS analysis of the corrosion by-products extracted from the inner-surface of a copper stagnated for 8 hours with synthetic water (DIC = 80 mg CaCO_3_/L; OCl^-^ = 8.0 mg Cl_2_/L).

To identify the elemental composition and targeting elemental chlorine at different layers of corrosion, by-products T-XRF was used. T-XRF of the corrosion by-products extracted from the pipe coupons by sonication revealed that chlorine was negligible in detached particles, despite that copper scales were removed (Figure 4a–b). These findings support the presence of an external/superficial film of malachite, and not chlorine-content scales. In contrast, after coupon sonication and surface scraping, chlorine was found as part of the removed scales. Interestingly, the mass of chlorine released as Cu-Cl corrosion by-product particles increased together with initial chlorine concentration in water. These results suggest that chlorine is probably forming part of non-superficial or “deeper” (from solution to the metal) corrosion by-product layers (Figure 4a–b). Additionally, coupons extracted from tested pipes were embedded in epoxy resin. To observe and analyze the thin scales formed on the copper inner surface, the resin was completely removed from the coupon surface, extracting with it the corrosion by-products formed over the metallic surface. EDS analysis of the scale attached to the resin surface (coupon face) reveals the presence of chlorine in the attached corrosion by-products (Figure 4c). These results confirm the presence of elemental chlorine in “deeper” corrosion by-products layers.

### 3.3. Chlorine Mass Balance

**Figure 5:**
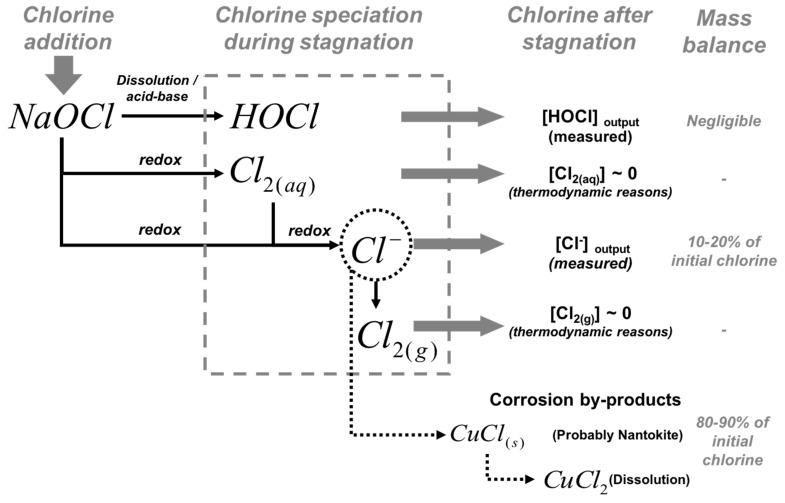
Speciation of free chlorine during stagnation. According to thermodynamic consideration and a mass balance of chlorine within the pipe during stagnation, most of the reduced chlorine precipitates as corrosion by-products, probably nantokite (CuCl).

Although free chlorine is completely reduced to chloride, no significant concentration of chloride was observed during the first hours of stagnation, probably because (1) chloride precipitates as copper corrosion by-products or/and (2) soluble copper-chloride complexes were formed (Figure 3). Figure 5 shows a schematic diagram of the free chlorine speciation during stagnation. According to thermodynamic considerations, surface characterization, and a complete mass balance of chlorine within the pipe during stagnation, between 80 to 90% of the reduced chlorine is captured in the pipe as corrosion by-products, probably as nantokite (CuCl) or forming soluble CuCl_2_^−^. Thermodynamic calculations indicate that soluble copper-chloride species represent less than 0.3% at pH = 7, and less than 0.5% at pH = 5 (maximum percentage for soluble copper-chloride species). These calculations suggest that chloride occurred as a solid corrosion by-product. Nantokite and atacamite have been the only corrosion by-products reported in copper corrosion studies in water [43], generally observed in pitting corrosion events. Nantokite dominates the speciation of Cu(I) for pH < 6 and is less soluble than cuprite. Atacamite is less soluble than cupric hydroxide or malachite, reported as the solid by-products that control copper solubility and dominate the speciation of Cu(II) between pH 5 and 9 [43] in new and aged pipe systems, respectively. Hence, free chlorine could be reduced to chloride and precipitated as nantokite, which is re-oxidized and covered by layers of malachite, cupric hydroxide, or other stable solid phases of Cu(II) that control the copper solubility and the release of copper into the water [44]. This is likely to occur in acidic microenvironments formed on the surface where the metallic copper oxidation takes place.

It is reasonable to assume that the chemical conditions in the solid-liquid interface are not necessarily the same that in bulk water. Close to the metallic surface, ion concentrations can be significantly higher than the bulk water and spatially heterogeneous across the pipe’s surface [45,46]. The Pourbaix diagram of solid corrosion by-products potentially formed over the metallic copper (Figure S4) in conditions of high ion concentration (HCO_3_^−^, Cl^−^, and low pH) predict the formation of cuprite or/and nantokite layers (depending on the pH) covered by passivating films of malachite, that are probably formed by cupric hydroxide ageing. 

Hence, our results strongly suggest that in new copper pipes systems, after 4–5 h of stagnation, the effective disinfection through free chlorine is lost due to corrosion.

## 4. Conclusions

The results show that the consumption of DO and free chlorine are processes that can be modeled as a first-order kinetic, but the chlorine constant depends on the initial concentration, probably due to diffusion processes. The free chlorine was depleted in 4–5 h due to corrosion, and from 80 to 90% of chlorine probably precipitates as chloride. In addition, our measurements reveal that after 8 h of stagnation, the presence of chlorine enhances copper release, while a decrease of copper concentration is noted at 4–5 h. After this time, high concentration of free chlorine has an opposite effect, probably due to the precipitation of nantokite. Surface analysis suggests that cuprite and nantokite are subsequently covered by layers of malachite, tenorite, and probably cupric hydroxide. Hence, the results of DO and chlorine kinetics, copper release, surface characterization, and thermodynamic calculations show that there is a loss of free chlorine from the bulk water due to corrosion. The loss of free chlorine is a serious disinfection issue, rather than the copper released into the water. These results prompt more attention of governmental organizations and further research in drinking water quality of copper premise plumbing.

## Supplementary Materials

The following are available online at https://www.mdpi.com/1996-1944/12/22/3676/s1, Figure S1: Copper release measurements in pipes with water at different chlorine concentration after 8 h of stagnation, Figure S2: Pipe tests conducted with different initial concentration of free chlorine, Figure S3: Scanning electron microscopy (SEM) images of new copper pipes in stagnant conditions with synthetic water (chlorine concentration of 4 mg/L, DIC of 80 mg as CaCO_3_, and pH 7.2), Figure S4: Pourbaix diagram of the corrosion by-products formed on the metallic surface of a copper-chlorine-carbonate system at 25 °C (respect to standard hydrogen electrode, SHE).
