# Supplementary material for: Chlorine Reduction Kinetics and its Mass Balance in Copper Premise Plumbing Systems During Corrosion Events
Source: Materials (Basel). 2019 Nov 8;12(22):3676. doi: 10.3390/ma12223676 (PMC6887719; doi:10.3390/ma12223676)
Supplement: Supplementary file 1 [file materials-12-03676-s001.pdf]

materials

Supplementary

Chlorine Reduction Kinetics and its Mass Balance in Copper Premise Plumbing Systems During Corrosion Events

Ignacio T. Vargas [1,2,*], Javiera M. Anguita [2], Pablo A. Pastén [1,2] and Gonzalo E. Pizarro [2]

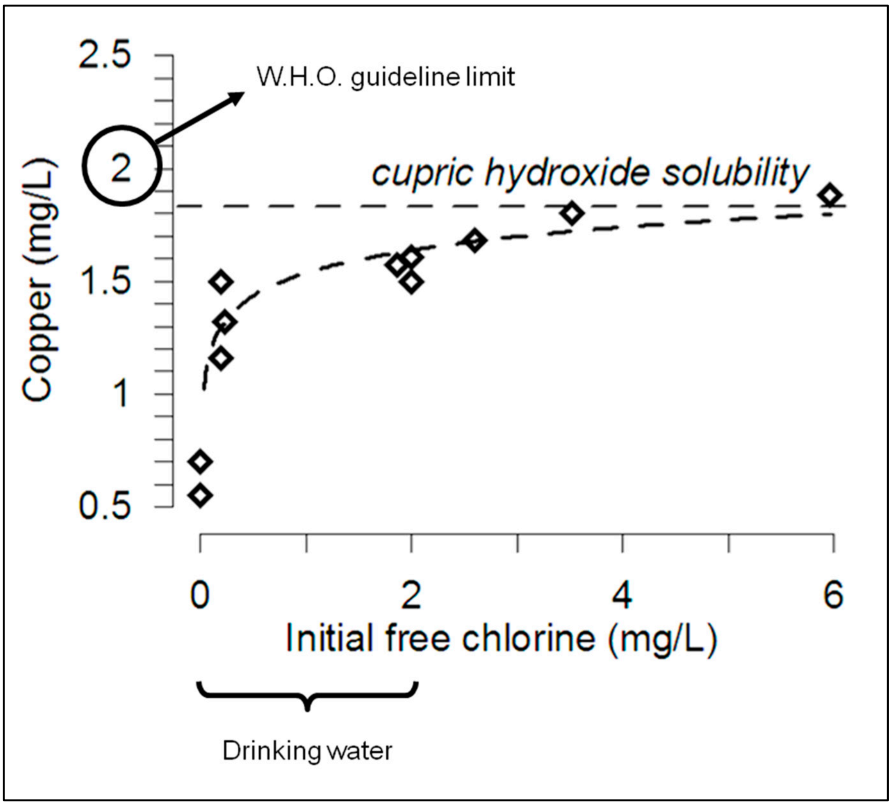

Figure S1. Copper release measurements in pipes with water at different chlorine concentration after 8 h of stagnation. Although copper release depends on the initial chlorine concentration, copper concentration is controlled by the solubility of corrosion by-products. According to thermodynamic calculations based on water chemistry parameters of the experiments, cupric hydroxide controls copper solubility at 1.8 mg/L. In all experiments, copper in water does not reach the W.H.O. guideline limit of 2 mg/L, in particular for typical chlorine concentrations in drinking water (0.2–2 mg/L).

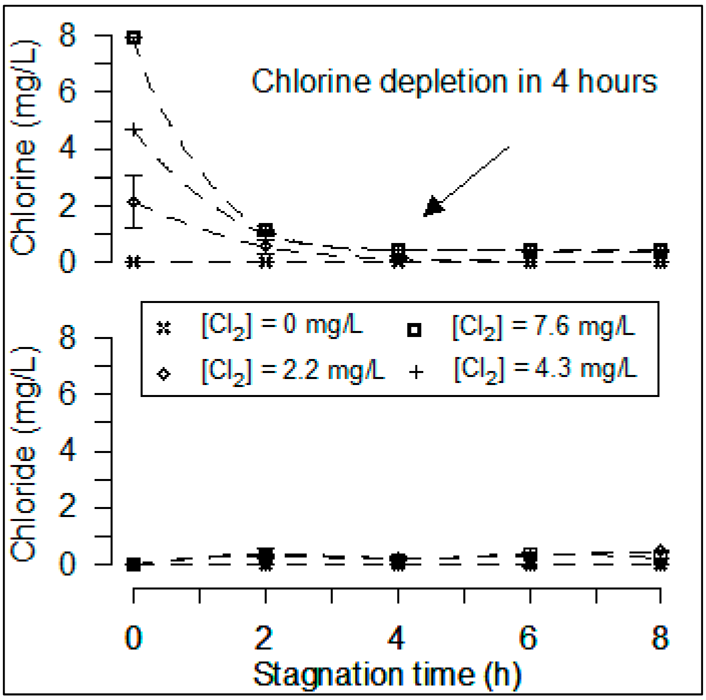

Figure S2. Pipe tests conducted with different initial concentrations of free chlorine. Results of chlorine consumption indicate that after approximately 4 hours, chlorine added with disinfection purposes is depleted. Measurements of chloride during stagnation suggest that most of the free chlorine is reduced to chloride precipitated during stagnation.

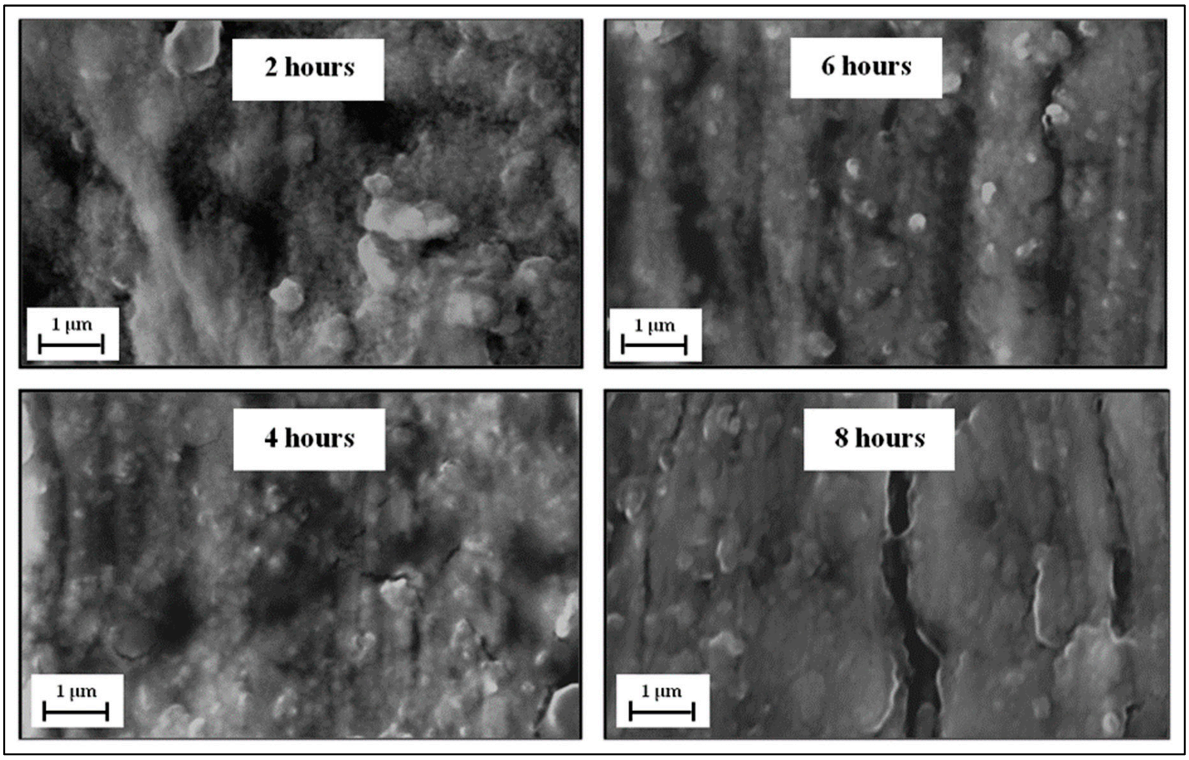

Figure S3. Scanning electron microscopy (SEM) images of new copper pipes in stagnant conditions with synthetic water (chlorine concentration of 4 mg/L, DIC of 80 mg as $CaCO_3$, and pH 7.2). Images show homogeneous superficial films (identified by GI-XRD as mainly malachite).

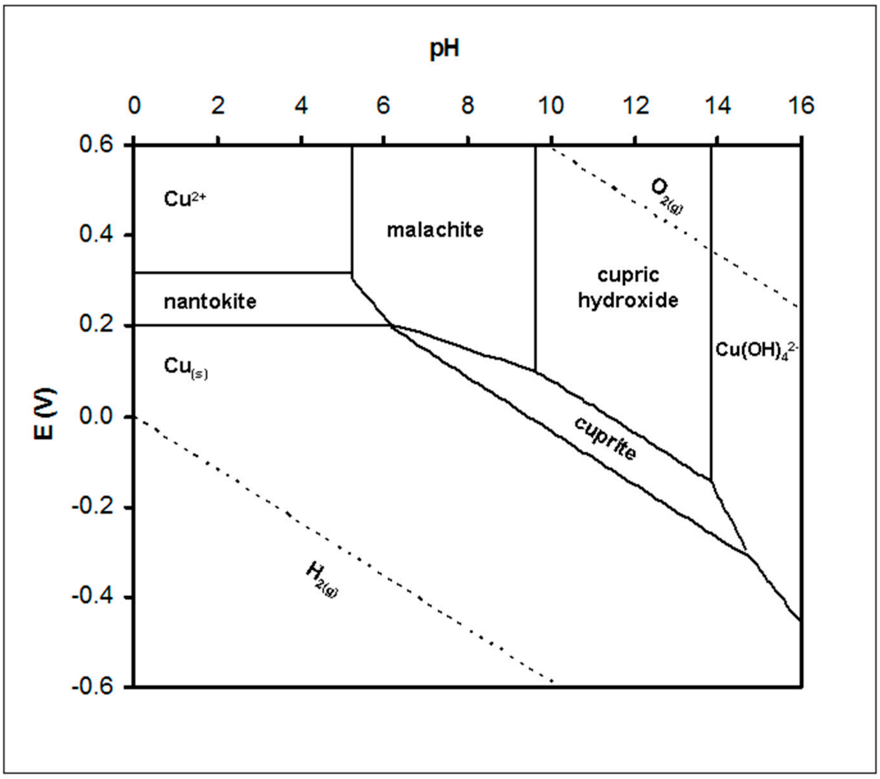

Figure S4. Pourbaix diagram of the corrosion by-products formed on the metallic surface of a copper-chlorine-carbonate system at 25 °C (respect to SHE). Concentrations increased 10^3 times in order to simulate high availability of hydrogen ion, chloride, and dissolved inorganic carbon at the metal-liquid interface.

© 2019 by the authors. Submitted for possible open access publication under the terms and conditions of the Creative Commons Attribution (CC BY) license (http://creativecommons.org/licenses/by/4.0/).
